# Enhancement of Radiation Sensitivity by Cathepsin L Suppression in Colon Carcinoma Cells

**DOI:** 10.3390/ijms242317106

**Published:** 2023-12-04

**Authors:** Ramadan F. Abdelaziz, Ahmed M. Hussein, Mohamed H. Kotob, Christina Weiss, Krzysztof Chelminski, Tamara Stojanovic, Christian R. Studenik, Mohammed Aufy

**Affiliations:** 1 Department of Pharmaceutical Sciences, Division of Pharmacology and Toxicology, University of Vienna, 1090 Vienna, Austria; ramadan.faried86@gmail.com (R.F.A.); kotobm84@univie.ac.at (M.H.K.); a01546450@unet.univie.ac.at (C.W.); mohammed.aufy@univie.ac.at (M.A.); 2 Division of Human Health, International Atomic Energy Agency, Wagramer Str. 5, 1400 Vienna, Austria; k.chelminski@iaea.org; 3 Programme for Proteomics, Paracelsus Medical University, 5020 Salzburg, Austria; tamara.stojanovich@gmail.com

**Keywords:** cancer, radiotherapy, cath L, knockout, inhibitor, colon carcinoma, radiation dose

## Abstract

Cancer is one of the main causes of death globally. Radiotherapy/Radiation therapy (RT) is one of the most common and effective cancer treatments. RT utilizes high-energy radiation to damage the DNA of cancer cells, leading to their death or impairing their proliferation. However, radiation resistance remains a significant challenge in cancer treatment, limiting its efficacy. Emerging evidence suggests that cathepsin L (cath L) contributes to radiation resistance through multiple mechanisms. In this study, we investigated the role of cath L, a member of the cysteine cathepsins (caths) in radiation sensitivity, and the potential reduction in radiation resistance by using the specific cath L inhibitor (Z-FY(tBu)DMK) or by knocking out cath L with CRISPR/Cas9 in colon carcinoma cells (caco-2). Cells were treated with different doses of radiation (2, 4, 6, 8, and 10), dose rate 3 Gy/min. In addition, the study conducted protein expression analysis by western blot and immunofluorescence assay, cytotoxicity MTT, and apoptosis assays. The results demonstrated that cath L was upregulated in response to radiation treatment, compared to non-irradiated cells. In addition, inhibiting or knocking out cath L led to increased radiosensitivity in contrast to the negative control group. This may indicate a reduced ability of cancer cells to recover from radiation-induced DNA damage, resulting in enhanced cell death. These findings highlight the possibility of targeting cath L as a therapeutic strategy to enhance the effectiveness of RT. Further studies are needed to elucidate the underlying molecular mechanisms and to assess the translational implications of cath L knockout in clinical settings. Ultimately, these findings may contribute to the development of novel treatment approaches for improving outcomes of RT in cancer patients.

## 1. Introduction

The worldwide burden of cancer escalated to 19 million new cases and 10 million deaths in 2019 [1]. Projections from the World Health Organization suggest that by 2030, there will be a rise in annual cancer incidence to 22 million and annual cancer deaths to 13 million [2].

Colorectal cancer ranks as the third most prevalent cancer in men and the second most frequently diagnosed cancer in women. There were up to 1.9 million new cases in 2020 [3].

Achieving successful cancer treatment requires a comprehensive and multifaceted approach. RT is an integral component of cancer treatment, alongside surgery and systemic therapy. Nearly half of all cancer patients are estimated to undergo RT as part of their treatment [4,5,6,7]. Over the past few years, significant progress has been made in RT, driven by advancements in radiobiology, advanced imaging techniques, and innovative treatment delivery approaches [8,9].

Imaging technique advancements, including image-guided radiotherapy (IGRT), intensity-modulated radiotherapy (IMRT), stereotactic body radiotherapy (SBRT), and proton beam therapy (PBT), have rapidly propelled the idea of precision radiotherapy [10,11]. These radiotherapy techniques allow for enhanced precision, reduced planning margins, and the administration of higher doses to the tumor, potentially leading to improved outcomes for patients [12,13].

RT is a widely utilized and highly effective treatment modality for cancer and aims to maximize DNA damage, initiating a series of events that could potentially result in cancer cell death, while minimizing damage to healthy cells [7,14].

Radiosensitization is an innovative method used in the delivery of RT for enhanced efficacy and reduced side effects. Radiosensitizers are chemicals or pharmaceutical agents that can enhance the killing effect on tumor cells by accelerating DNA damage and producing free radicals indirectly during RT treatment [15,16].

Radiosensitizers were classified into five categories: (1) Suppression of intracellular thiols or other endogenous radioprotective substances; (2) Formation of cytotoxic substances by radiolysis of the radiosensitizer; (3) Inhibitors of repair of biomolecules; (4) Thymine analogs that can incorporate into DNA; (5) Oxygen mimics that have electrophilic activity [15].

When cells are exposed to ionizing radiation, genomic DNA faces direct damage from secondary electrons and/or indirect damage from reactive oxygen species (ROS), resulting in single-strand breaks (SSBs) and double-strand breaks (DSBs) [17,18]. The DNA damage response (DDR) comes into play as cells react to genomic DNA damage, especially DSBs, through the recruitment of various factors within the intricate DDR network, initiated within min of DNA damage occurrence [19,20,21].

DSBs’ repair mechanisms involve two pathways: the efficient yet error-prone non-homologous end-joining (NHEJ) pathway, and the less efficient but more precise homologous recombination (HR) pathway [22,23,24]. NHEJ operates in the G1 phase, joining free ends with an increased risk of mutagenesis. Activation of the NHEJ involves recruiting the phosphoinositol-kinase-kinase-kinase (PI3K) ataxia telangiectasia mutated (ATM) to DSB sites, leading to H2A.X phosphorylation (γH2AX) near break sites. This phosphorylation propagates over kilobase distances, forming γH2AX DNA damage foci [25]. These foci act as beacons, recruiting downstream repair and effector proteins to damage sites, activating the G1 cell cycle checkpoint kinase Chk1 and inducing cell cycle arrest in a p53-dependent manner [26]. Conversely, HR, occurring during G2 and S phases, relies on a template sister chromatid for precise DSB alignment. HR-associated phosphorylation of H2A.X necessitates PI3K ataxia telangiectasia related (ATR), recruiting key proteins to the replication fork for accurate DNA template repair [22,27,28]. Unrepaired or misrepaired DSBs can lead to genomic instability, prompting either cell death or cellular senescence (an irreversible cell cycle arrest state) [19,29]. In tumor cells, exposure to ionizing radiation (IR) incites DNA damage, leading to a spectrum of cell death mechanisms, including apoptosis, necrosis, autophagic cell death, and mitotic catastrophe [27,29].

The efficiency of DNA repair mechanisms is a key contributor to radiation resistance in cells. Cells with proficient DNA repair pathways can fix these breaks, reducing the lethal effects of radiation. This heightened ability to repair DNA damage enables the survival of cancer cells and contributes significantly to their resistance to RT [30,31,32]. Radiation takes advantage of the susceptibility of cancer cells with compromised DNA repair mechanisms, in contrast to normal cells that efficiently repair double-stranded breaks. Utilizing a fractionated approach involves dividing the total radiation dose into multiple daily treatments. [33,34]. Fractionation reduces both immediate and delayed toxicity in normal tissue by leveraging its superior DNA repair capabilities between fractions [35,36]. Additionally, fractionation facilitates reoxygenation and the redistribution of the cell cycle between fractions, enhancing the sensitivity of tumors to radiation [37,38].

Despite notable advancements in the molecular mechanisms of carcinogenesis and modern RT techniques, and the success of RT, whether administered alone or in combination, there is a primary challenge; the radioresistance exhibited by cancer cells due to DNA damage repair system activity after radiation exposure. Therefore, elevating the radiosensitivity of cancer cells enhances the mechanisms that lead to their elimination during RT, thereby extending the survival of cancer patients [39].

More recently, studies have identified caths, a family of lysosomal peptidases, as potential key contributors to the tumor microenvironment during RT [40]. They play a crucial role in breaking down proteins internalized in lysosomes through processes such as endocytosis, phagocytosis, and autophagocytosis and involved in apoptosis regulation [41,42].

While ionizing radiation (IR) serves as a significant source of external reactive oxygen species (ROS) and plays a crucial role in the internal generation of ROS within the body [43], another study explored the relationship between ROS, thioredoxin reductase (TrxR) inhibition, and cath L in neuronal cells. The findings suggested that TrxR inhibition-induced oxidative stress disrupts cath L processing, affecting its pro-autophagy function [44].

In addition, Thirusangu et al. [45], demonstrated that the antimalarial quinacrine (QC) induces autophagy in ovarian cancer cells, with cath L playing a crucial role in promoting QC-induced autophagic flux and apoptotic cell death. In a separate study, results showed a distinct cytotoxic mechanism in cervical cancer cells induced by resveratrol (RSV), where cathepsin L (cath L) serves as a death signal integrator [46].

Lately, there has been a growing focus on the role of lysosomes, particularly lysosomal peptidases, in radioresistance. Previous studies demonstrated the radioresistant function of caths. Zhang et al., 2018 [47], investigated the role of cathepsin B, a significant member of the cysteine peptidases, in the radioresistance of glioblastoma cell lines by enhancing homologous recombination. Furthermore, the knockdown of cath B triggered a cell cycle arrest in the G0/G1 phases, leading to a consequential impairment in homologous recombination efficiency and, consequently, impacting radiosensitivity. Moreover, the study suggested the role of cathepsin B in facilitating radiation resistance in colon carcinoma cell lines [48]. Another study showed radiation triggers cathepsin S expression through ROS-IFN-gamma pathways, and this heightened expression may play a role in radioresistance [49]. In addition, earlier research investigated whether suppression of cath L enhances radiosensitivity in human glioma U251 cells through G2/M cell cycle arrest and induction of DNA damage [50]. These effects can ultimately impact the efficacy of RT and contribute to the development of treatment resistance [23].

In this study, our objective was to explore the contribution of cath L, in the development of radiation sensitivity. Additionally, we investigated the effects of a specific cath L inhibitor and the knockout of cath L using CRISPR/Cas9 technology on radiosensitization in colon carcinoma cells (caco-2). Our experimental findings demonstrated that the knockdown of cath L led to a significant increase in radiosensitivity.

## 2. Results

### 2.1. Expression of Cath L after Exposure to Ascending Doses of Ionizing Irradiation

To investigate the effect of ionizing radiation on the expression level of cath L, caco-2 cells were exposed to ascending doses of the ionizing radiation. Cells were then harvested, subjected to protein extraction, SDS-PAGE under reducing conditions, and immunoblotting using specific antibodies to cath L.

**Figure 1:**
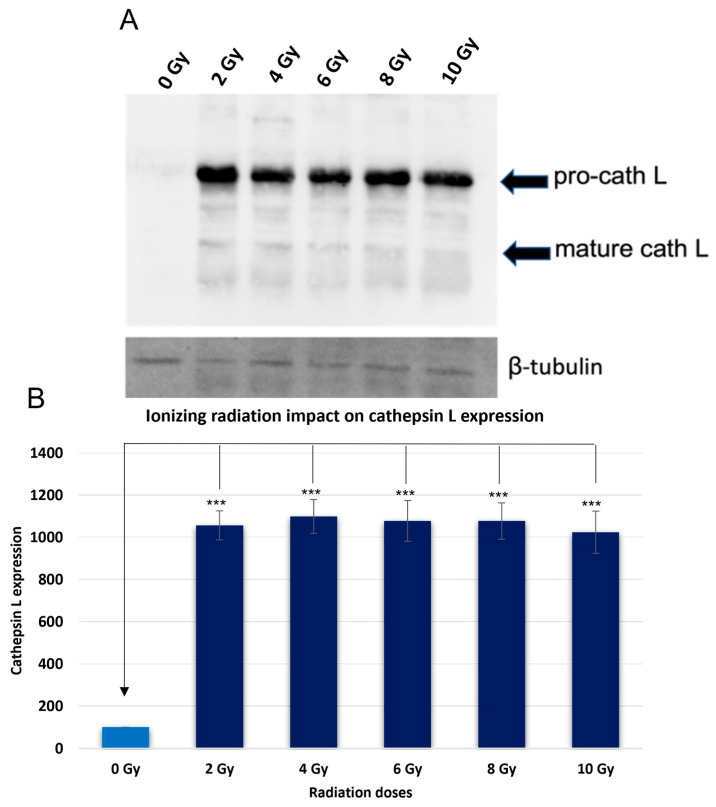
Assessment of cath L expression under varying radiation doses. (**A**) Western blot was performed to investigate the regulation differences. (**B**) The cellular cath L levels were compared with and without radiation treatment, and statistical analysis was performed using the *t*-test with GraphPad Prism software. The results indicated a highly significant difference (*** *p* < 0.001), and the graphs represent the mean ± standard error (SE) with a sample size of N = 5. (0 Gy = Control).

The investigation into the effect of ionizing radiation on cath L expression in caco-2 cells revealed intriguing findings. Upon exposure to increasing doses of ionizing radiation (2 Gy, 4 Gy, 6 Gy, 8 Gy, and 10 Gy), a substantial upregulation of cath L expression was observed (Figure 1). In contrast, control cells, which were not subjected to radiation, exhibited very low levels of cath L expression.

Significantly, the increase in cath L expression seemed to occur irrespective of the radiation dose, as the levels remained relatively consistent across all doses of irradiation. This observation implies that ionizing radiation induces a dose-independent upregulation of cath L in caco-2 cells.

**Figure 2:**
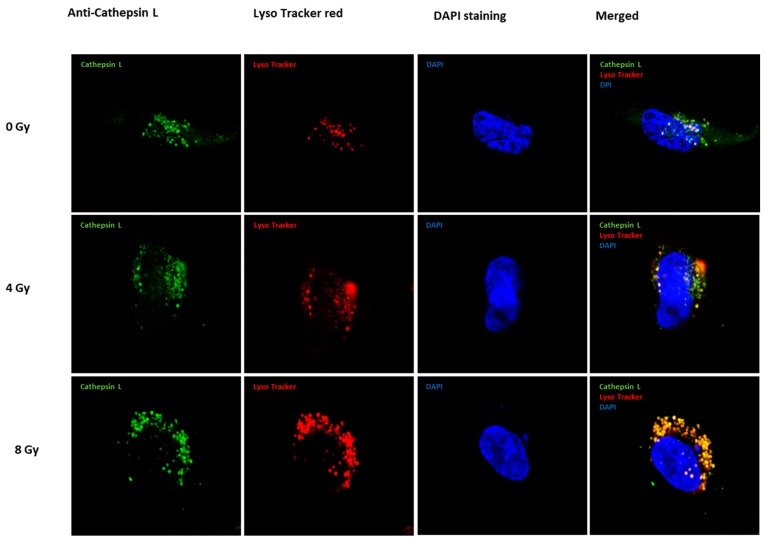
Cath L localization in irradiated and non-irradiated (control) caco-2 cells (63× magnification). Application of anti-cath L antibody (green) shows the lysosomal and membrane-bound cath L localization, LysoTracker red for lysosomal staining and 4′,6-diamidino-2-phenylindole (DAPI) visualizes nuclear DNA (blue) in fixed cells.

Aligned with this line of evidence, immunofluorescence results validated the upregulation of cath L (depicted in green fluorescence) induced by irradiation treatment in caco-2 cells (Figure 2). The heightened fluorescence signals described the increased expression, emphasizing the impact of irradiation on elevating cath L levels. Furthermore, parallel results are observed with LysoTracker red, employed for lysosomal staining, confirming consistent localization patterns. The merged figures provide a comprehensive visual representation, highlighting the significance of the expression in yellow/orange-reflecting lysosome localization and cath L.

### 2.2. In Vitro Labeling of Cysteine Cathepsins and Pull-Down of Cath L in Irradiated and Non-Irradiated Caco-2 Cells

The investigation aimed to assess the accessibility of the active sites of cysteine caths to the activity-based probe DCG04. Originally designed as a selective activity-based probe for cysteine peptidases [51], DCG04 was used to probe the active sites of cysteine caths in caco-2 cells. Prior to western blot analysis of cellular extracts with streptavidin-horseradish peroxidase, the cells were exposed to five different ionizing irradiation doses. The negative control included non-irradiated caco-2 cells.

**Figure 3:**
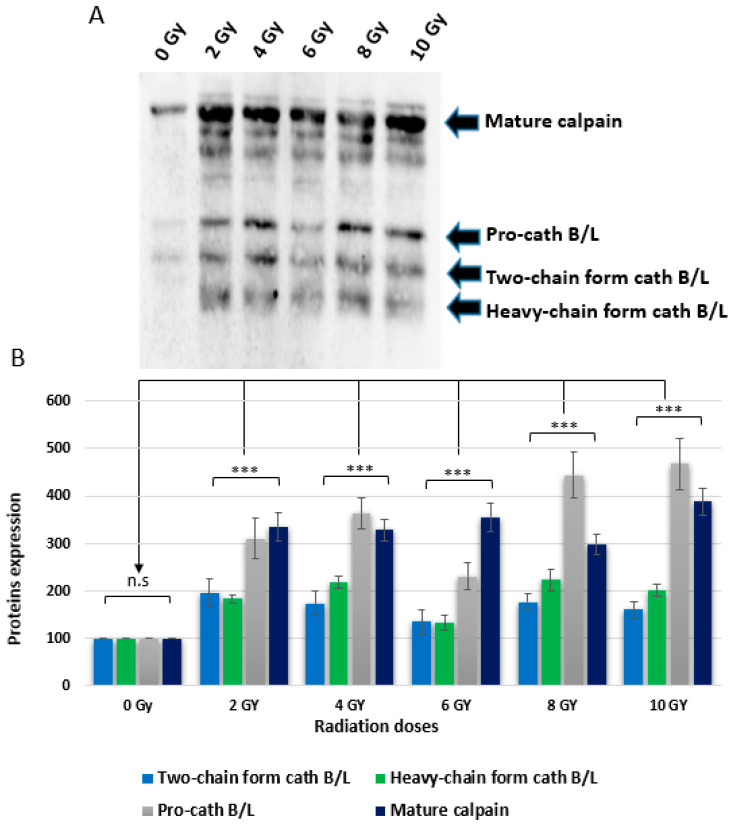
Impact of radiation treatment on the labeling of cysteine cathepsins’ active sites using DCG04. (**A**) Active-site labeling with DCG04 was carried out, and protein samples underwent electrophoresis and subsequent western blotting using streptavidin-horseradish peroxidase. (**B**) The cellular content of procathepsin B/L, heavy chain-form cath B/L, and mature form (two-chain form) of cath B/L in irradiated caco-2 cells labeled with DCG-04 bands was compared to cellular content from control cells labeled with DCG-04 only, non-significant (n.s). The data underwent analysis using a two-way ANOVA followed by Dunnett’s post hoc analysis. Statistical calculations were performed using GraphPad Prism. The results indicated a highly significant difference (*** *p* < 0.001), and the graphs represent the mean ± standard error (SE) with a sample size of N = 5. (0 Gy = Control).

Upon conducting the experiment, four distinct bands were observed in the DCG04-labeled cells. The higher band had an approximate molecular weight of 70 kDa, which co-migrates with the processed form of calpain 1 or 2; another was approximately 40 kDa, which co-migrates with the unprocessed form of cath B or cath L. A third band had approximate molecular weight 30 kDa, which co-migrates with the heavy-chain form of cath B or cath L. The last band was about 25 kDa, which co-migrates with the two-chain form of cathepsin B or cath L. These findings substantiate that ionizing irradiation prompts the upregulation of various cysteine peptidases, specifically, cathepsin B and cathepsin L (Figure 3).

**Figure 4:**
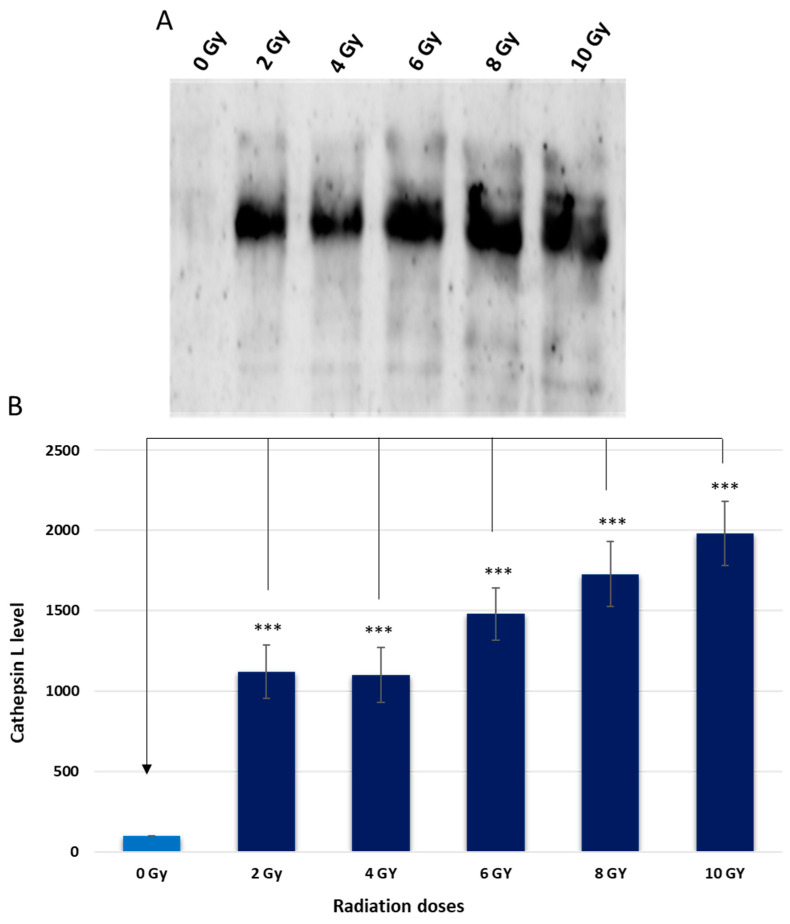
Avidin pull-down experiment of cysteine cathepsins with and without radiation treatment. (**A**) Conjugated proteins to avidin sepharose beads were subjected to SDS-PAGE and western blotting with antibodies specific to human cath L. (**B**) Ratio of cellular contents of cath L with and without radiation treatment. The data were analyzed by *t*-test and statistics using GraphPad Prism. *** *p* < 0.001. Graphs are shown as mean ± SE, N = 5. (0 Gy= Control).

To identify some of those bands, a pull-down experiment was conducted using streptavidin–sepharose beads. The proteins that bound to the streptavidin–sepharose beads were then analyzed through protein SDS-PAGE and western blotting, using antibodies against cath L (Figure 4). The results of the pull-down experiments, which aimed to investigate the interactions of cysteine caths with DCG04 in a cellular content, provided further validation and reinforcement to the findings from earlier in vitro labeling studies. Moreover, the investigation revealed a notable effect on cath L levels within the cells when subjected to various doses of ionizing irradiation which led to a significant upregulation of cath L expression, indicating that this peptidase might play a crucial role in the cellular response to radiation treatment.

### 2.3. Unraveling the Impact of Cath L Inhibition on Ionizing Irradiation-Induced Cytotoxicity in Caco-2 Cells

**Figure 5:**
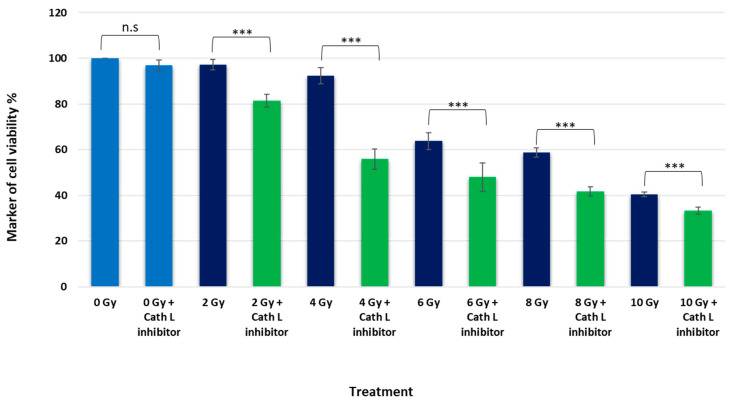
Cytotoxicity analysis of treated cells by radiation and a cath L inhibitor, compared to untreated cells. Statistical analysis was carried out using a *t*-test with GraphPad Prism, and the results indicated significant differences (*** *p* < 0.001), non-significant (n.s). The graphs represent the mean values along with the standard error (SE), and the study had a sample size of N = 8. (0 Gy = Control).

To investigate the role of cath L in ionizing radiation-induced cytotoxicity, experiments were conducted using caco-2 cells. Two groups of the cells were exposed to escalating doses of ionizing radiation, with and without inhibition of cath L using the cath L-specific inhibitor Z-FY(tBu)DMK. The radiation cytotoxicity was assessed, and intriguingly, it was observed that the cytotoxic effects were dose-dependent, while higher doses led to higher cytotoxicity than lower doses. However, what is particularly noteworthy is that the cytotoxic effects of ionizing radiation were significantly enhanced when cath L was inhibited, as depicted in Figure 5.

For instance, radiation treatment at 2 Gy induced only a slight cytotoxic effect, but this effect increased by approximately 20% when the same dose was administered upon cath L inhibition. Similarly, radiation treatment at 4 Gy resulted in a cell viability reduction of less than 10%, while at the same dose with cath L inhibition, cell viability was decreased by more than 40%. At 6 Gy, cell viability was reduced by approximately 32%, which was further decreased to over 50% after cath L proteolytic activity was eliminated. At 8 Gy, cell viability was reduced by about 42%, but this inhibition was increased to 60% upon cath L inhibition. Lastly, irradiation at 10 Gy reduced cell viability by approximately 60%, and this reduction increased to more than 70% upon cath L inhibition.

### 2.4. Cath L Knockout by CRISPR/Cas9

**Figure 6:**
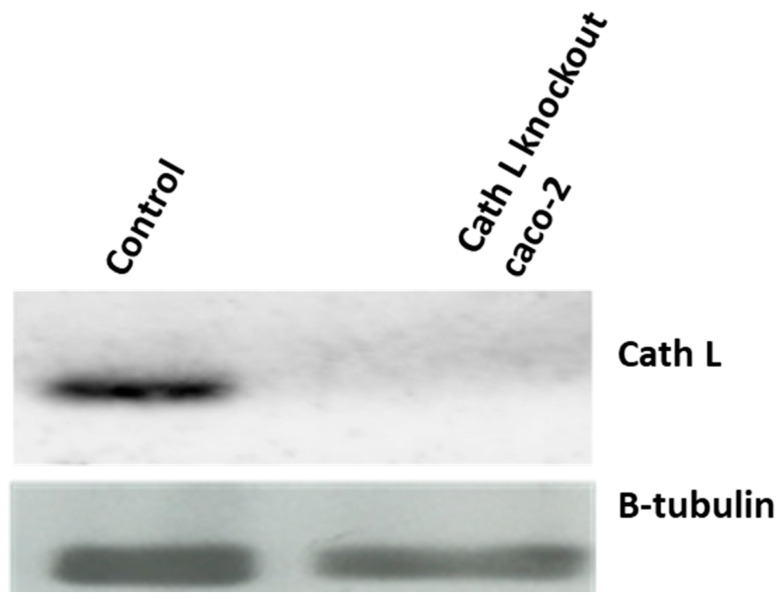
Examination of cath L expression in both control and cath L knockout cells.

Cath L knockout caco-2 cells were prepared following the methods described in the Section 4 and were exposed to the same doses of ionizing radiation as used in previous experiments. Caco-2 cells, transfected with CRISPR/Cas control plasmid, were used as a control group. The transfection efficiency was confirmed by harvesting proteins from both cath L knockout and control cells. These harvested proteins were subjected to SDS-PAGE and immunoblotting using antibodies specific to human cath L. Cath L knockout cells showed no expression of cath L, while control cells exhibited clear expression, as presented in Figure 6.

**Figure 7:**
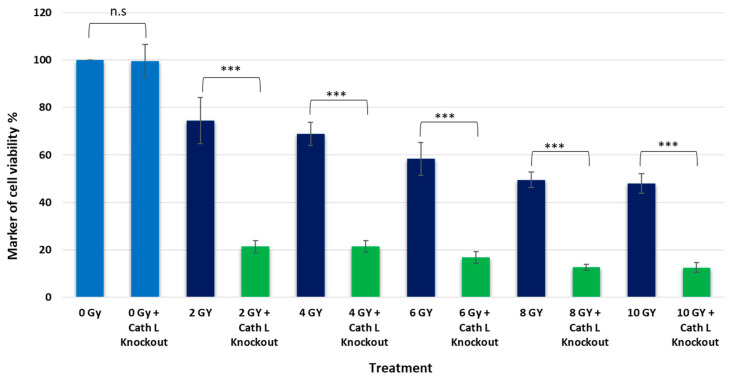
Evaluating the effect of cath L knockout on cellular toxicity in both irradiated and non-irradiated cells. Statistical analysis was performed using a *t*-test with GraphPad Prism, revealing a highly significant difference (*** *p* < 0.001), non-significant (n.s). The graphs depict the mean values along with the standard error (SE), and the study had a sample size of N = 8. (0 Gy = Control).

Interestingly, the impact of ionizing radiation on cath L knockout cells was significantly more pronounced compared to the previous experiment. For instance, at 2 Gy dose, caco-2 cells treated with cath L inhibitor exhibited approximately 20% cell mortality, whereas cath L knockout cells reached about 80% mortality. At other doses, the mortality in cath L knockout cells was much higher compared to control cells (Figure 7). These results provide additional support to our previous data, suggesting that cath L plays a vital role in attenuating the cytotoxic effects of ionizing irradiation.

### 2.5. Caspase 3/7 Activity Assay upon Ionizing Irradiation in the Presence and Absence of Cath L Inhibitor

To explore the influence of cath L on cell apoptosis in caco-2 cells under ionizing radiation, we exposed the cells to escalating doses of ionizing irradiation. This exposure was carried out in both the presence and absence of cath L inhibitor.

In the non-radiated cell population, the impact of cath L inhibition on caspase 3/7 activity was found to be minimal. However, as radiation doses increased, a dose-dependent elevation in caspase 3/7 activity became evident. Specifically, at 2 Gy, caspase 3/7 activity increased by 26% ± 2.75 compared to non-radiated cells. Interestingly, when cath L inhibition was introduced at the same dose, the activity further rose by 39% ± 1.3 relative to the control group. This indicates an additional 13% increase attributed to cath L inhibition above the heightened activity induced in non-radiated cells.

Likewise, at 4 Gy, caspase 3/7 activities increased by 50.1% ± 2.3 compared to non-radiated cells. With cath L inhibition at this dose, the activity escalated even more, reaching 66% ± 1.65 compared to the control group. This highlighted a significant 15.9% elevation in caspase 3/7 activity due to cath L inhibition.

This pattern continued with increased doses (6 Gy, 8 Gy, and 10 Gy), with cath L inhibition consistently augmenting caspase 3/7 activity compared to non-radiated cells. These findings suggest a potential regulatory role of cath L in influencing apoptotic signaling under escalating ionizing radiation conditions.

## 3. Discussion

Numerous studies have documented an increase in the expression levels of certain lysosomal caths following ionizing radiation exposure. These investigations have consistently observed an upregulation of these caths, highlighting a potential link between ionizing radiation and the cellular response involving lysosomal cathepsin activity. This phenomenon has been the subject of interest in various research studies aiming to understand the molecular mechanisms underlying the cellular response to ionizing radiation, and its implications for cellular homeostasis and DNA damage repair. It was found that cathepsin S was upregulated upon RT in different tumors; this upregulation plays an important role in cell resistance to RT [49]. In our study, the upregulation of cath L in caco-2 cells in response to ionizing irradiation exposure was confirmed by different methodologies. This involved evaluating cath L expression using immunoblotting techniques with specific cath L antibodies (Figure 1).

In addition, the heightened fluorescence signals observed in the immunofluorescence results serve as a compelling indicator of the increased expression of cath L following radiation treatment in caco-2 cells (Figure 2). This heightened expression underscores the notable impact of radiation on elevating cath L.

This can have a profound impact on shaping the cellular response, potentially affecting various aspects of cellular behavior, signaling pathways, and ultimately, the overall homeostasis and functionality of the cell.

These findings align with the outcomes of a prior study conducted by Zhang et al., (2015) [52], focusing on glioma U251 cells, thereby providing additional support and contextual relevance to the observed phenomenon.

The parallel results obtained by LysoTracker red, a marker specifically employed for lysosomal staining, contribute to the robustness of the findings. The confirmation of consistent localization patterns adds an extra layer of reliability to the study. Lysosomes play a crucial role in cellular degradation processes, and the correlation between cath L expression and lysosomal localization implies a potential connection between the upregulated cath L and cellular degradation pathways. However, there is a disparity in the levels of cath L between the western blot and immunofluorescence results, which may be attributed to mitophagy. A study by Yanxian et al. (2023) [53] previously demonstrated mitophagy in cells exposed to ionizing radiation, showing a dose-dependent increase in mitophagy. As a result, our hypothesis proposes that the intensified lysotracker red staining in the immunofluorescence images is not solely due to elevated specific cathepsin expression. Instead, it likely stems from an enlarged lysosomal area, as indicated by lysotracker red, resulting from the incorporation of mitochondrial debris within lysosomal compartments. Therefore, the combination of heightened fluorescence signals, confirmation through LysoTracker red staining, and the visual representation of merged figures collectively strengthens the evidence for the impact of irradiation on cath L expression and its localization within lysosomes.

Additionally, the proteolytic activity of cysteine peptidases (in particular, cath L) was examined using the activity-based probe DCG-04 followed by pull-down of cysteine peptidases and immunodetection of cath L (Figure 3 and Figure 4). These combined approaches provided solid evidence and confirmed the observed increase in both expression and activity of cath L in response to ionizing irradiation in caco-2 cells. Interestingly, the resistance to radiation exposure was decreased significantly after inhibition of the proteolytic activity of cath L by cath L-specific inhibitor Z-FY (tBu)DMK or by knocking out cath L expression via CRISPR/CAS 9 (Figure 6 and Figure 7). The inhibition was somewhat greater in cath L knockout cells compared to cath L inhibited cells. Our speculation is that the cath L inhibitor does not eradicate the entire proteolytic activity of cath L, while CRISPR/CAS9 completely abolishes the expression of cath L.

**Figure 8:**
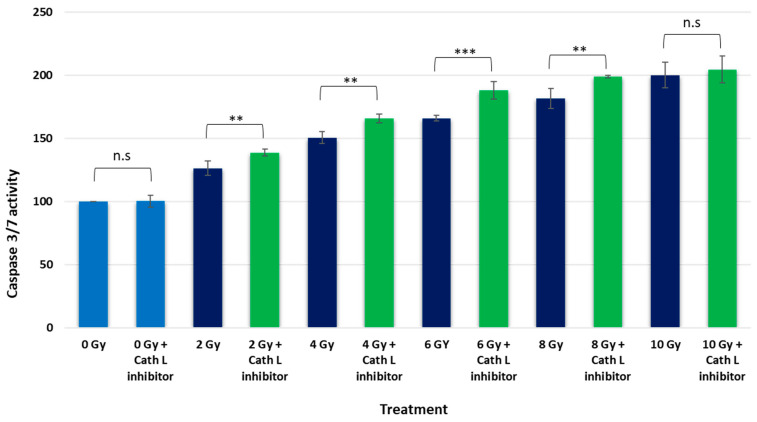
Assessing the influence of cath L inhibition on apoptosis rates in cells exposed and non-exposed to ionizing radiation via caspase 3/7 activity assay. The data underwent analysis using a two-way ANOVA followed by Dunnett’s post hoc analysis, revealing significance levels (** *p* < 0.01; *** *p* < 0.001; N = 4), non-significant (n.s), (0 Gy = Control). Statistical calculations were carried out using GraphPad Prism.

Other investigations reported that the expression of cathepsin B was upregulated in glioblastoma to provide resistance to RT by enhancing the homologous recombination [47]. In this investigation, it was found that the expression of cath B was elevated almost four-fold after exposure to 4 Gy ionizing irradiation. In our study, the expression of cath L was elevated about 10-fold when the cells were exposed to the same strength of ionizing radiation dose (Figure 1). In the same study [47], it was found that cath B knockdown glioblastoma cells showed a higher level of apoptosis compared to wild type glioblastoma cells. While, in our study, we found that the activities of caspase 3/7 were significantly elevated for most of the radiation doses in cath L-inhibited caco-2 cells (Figure 8). However, the elevation at 10 Gy was not significant. Our speculation is that, according to the previous literature, the small or intermediate radiation doses induce apoptosis while higher irradiation doses induce necrosis [54]. Radiation can induce apoptosis by both intrinsic and extrinsic pathways [55]. In the intrinsic pathways, radiation seems to induce apoptosis via a caspase-dependent pathway [56]. This is in agreement with our findings, in which caspases 3 and 7 showed higher activities in ionizing radiation-exposed caco-2 cells.

RT can alter mitochondrial membrane permeability through a caspase-dependent intrinsic pathway, increasing and releasing proapoptotic factors into the cytoplasm, thereby triggering a series of apoptotic cascades [57].

Collectively, these findings strongly indicate that the presence of cath L proteolytic activity renders cells less susceptible to ionizing irradiation. Moreover, a recent study investigated similar results on cath B and its involvement in mediating radiation resistance in caco-2. This correlation supports our current study, as we utilized the same cell line, reinforcing the validity and relevance of our research [48].

In conclusion, our study demonstrated cellular response to ionizing radiation, specifically highlighting the pivotal role of cath L in radioresistance. The observed upregulation and increased activity of cath L present potential avenues for further exploration in understanding the complex interplay between lysosomal dynamics, cellular degradation pathways, and responses to ionizing irradiation. In addition, our study involved the utilization of a specific cath L inhibitor and the application of CRISPR/Cas9 technology to knockout cath L in caco-2, which demonstrated a significant suppression in cath L expression, leading to increased radiosensitivity and cell death. We also assume that this effect is synergistic, reflecting an interplay between cathepsin L inhibition and radiation treatment. These findings may contribute to the development of clinical studies and targeted therapeutic approach strategies for enhancing the efficacy of RT in cancer treatment.

## 4. Materials and Methods

### 4.1. Cell Culture

Caco-2 cells were used in the study (these cells were given by Dr. Rosa Lemmens-Gruber, Vienna university). The cell line was propagated in Dulbecco’s Modified Eagle Medium (DMEM, Gibco, ThermoFisher Scientific, Waltham, MA, USA, CAS, 21063029), supplemented with 4 mM glutamine and 5% FBS (Sigma Aldrich, St. Louis, MO, USA, 9048-46-8). Antibiotics 100 units/mL penicillin and 100 μg/mL streptomycin (ThermoFisher Scientific, Waltham, MA, USA) were added, and cells were incubated at 37 °C in a 5% CO_2_ incubator [58,59].

### 4.2. Radiation Treatment

**Figure 9:**
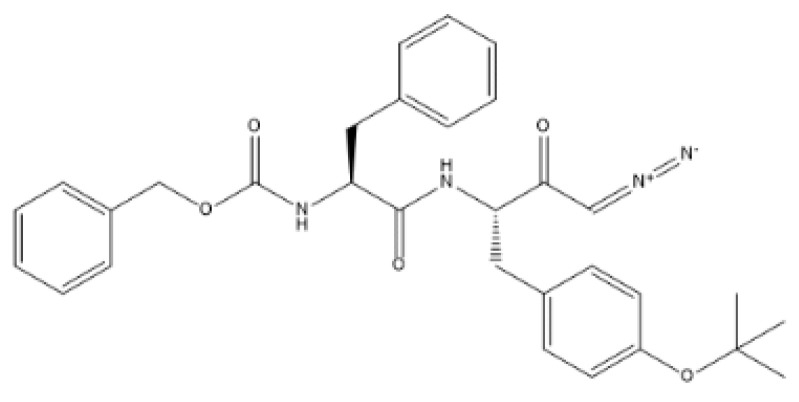
Z-Phe-Tyr(tBu)-diazomethylketone is an irreversible cathepsin L inhibitor, and this compound is commonly utilized as a selective inhibitor for cath L. It is a modified peptide derivative consisting of the amino acids phenylalanine (Phe) and tyrosine (Tyr), along with a diazomethylketone functional group attached to the tyrosine residue. The tBu abbreviation indicates the presence of a tert-butyl group, which enhances stability and influences the compound’s properties. Chemical Formula: C_31_H_34_N_4_O_5_.

Two sets of caco-2 cells underwent distinct treatments in this study. The first group was subjected to irradiation using a 6 MV photon beam from a medical linear accelerator (LINAC) by Varian Medical Systems, Palo Alto, CA, USA. These cells were divided into subgroups; one group was treated with cath L inhibitor (Z-FY(tBu)DMK, MedKoo Biosciences, Morrisville, NC, USA, CAS (114014-15-2) (Figure 9), and another group was not treated with this inhibitor. These groups received varying doses (2, 4, 6, 8, and 10 Gy) at a consistent dose rate of 3 Gy/min. The Eclipse treatment planning system (TPS) by Varian facilitated the precise assessment of the delivered doses [60,61].

Simultaneously, another set of caco-2 cells, treated and untreated with the cath L inhibitor, were not exposed to radiation and served as the negative control. In preparation for radiation exposure, approximately 10^7^ caco-2 cell lines underwent a 24 h treatment at 37 °C in a complete medium containing 10 μM (Z-FY(tBu)DMK). For the untreated groups, a solvent of Dimethylsulphoxide (final concentration 0.1%) was introduced. All samples were incubated at 37 °C in a 5% CO_2_ incubator for 24 h before protein harvesting and other upstream analysis.

### 4.3. Western Blotting

Initially, the cells were cultured in 100 mM cell culture dishes in a 5% CO_2_ incubator using DMEM medium supplemented with 5% fetal bovine serum. After aspirating the medium, the cells were washed twice with PBS. Subsequently, the cells were scraped and transferred to 1.5 μL centrifuge tubes and lysed in a lysis buffer (composed of 200 mM sodium acetate, 150 mM NaCl, pH 5.5, and supplemented with 40 μM E-64). The homogenization of cells was performed on ice using ultrasonication, followed by the addition of 0.1% Triton X-100. The homogenized cells were then incubated on ice for 30 min and cleared by centrifugation at 15,000× *g* for 10 min.

Next, the proteins were separated by 12.5% SDS-PAGE under reducing conditions and then transferred to a nitrocellulose membrane using semi-dry blotting at 25 V for 30 min. To block unspecific binding sites, the membrane was incubated for 3 h in a blocking solution (3% BSA Sigma Aldrich, St. Louis, MO, USA, CAS 9048-46-8 in PBS). The membrane was then incubated for 90 min with primary antibodies, including cath L (ThermoFisher Scientific, Waltham, MA, USA) CAS (404111), and β-tubulin (Sigma Aldrich, St. Louis, MO, USA, CAS, 32160707) (1:3000). After incubation, the membrane was washed five times with PBST and then incubated for another 90 min with the corresponding secondary antibodies, followed by three washes with PBST and one wash with PBS [62,63,64].

For visualization, enhanced chemiluminescence (Amersham ECL plus Western blotting detection reagent, GE Healthcare, Vienna, Austria) was used. The membranes were exposed to imaging system (ChemiDooc) Biorad, Hercules, CA, USA. The figures were quantified using ImageJ, 1.54f (NIH, Bethesda, MD, USA).

### 4.4. Fluorescent Immunocytochemistry Detection and Microscopy

Caco-2 cells were grown on Poly-D-Lysine (PDL; Gibco; ThermoFisher Scientific, Waltham, MA, USA, A38904-01)-coated glass cover slips and underwent exposure to ascending doses of ionizing radiation (4 Gy, 8 Gy, and 10 Gy) in addition to control samples. Cells were then preincubated with LysoTrackerred DND-99 (Thermo Fisher Scientific; L7528) for 30 min at room temperature, according to manufacturer’s instructions. Cells were washed with phosphate buffered saline (PBS) containing 0.03% sodium azide (NaN_3_) 2 × 5 min at room temperature and fixed with 4% paraformaldehyde (PFA) in PBS pH 7.4 for 30 min at RT. Cells were washed with PBS 2 × 5 min at room temperature and permeabilized with 0.3% TritonX-100 (Promega; H5141) in PBS pH 7.4 for 10 min at room temperature. Furthermore, cells were incubated with 1% Bovine Serum Albumin (BSA; Santa Cruz Biotechnology, Inc., Dallas, TX, USA; sc-2323) and 1% normal goat serum (NGS; Sigma-Aldrich; G9023) in PBS pH 7.4 for 2 h at room temperature and subsequently incubated with primary anti-cathepsin B antibody (rat anti-mouse/human MAB965), anti-cathepsin D antibody (goat anti-mouse AF1029), and anti-cath L antibody (mouse anti-human BMS166) overnight at 4 °C. Cells were washed with PBS 2 × 5 min at room temperature and incubated with following secondary antibodies: Alexa Fluor 488 anti-mouse IgG Fab2 (Molecular Probes, Eugene, OR, USA; 4408S), Alexa Fluor 647 anti-mouse IgG Fab2 (Molecular Probes; 4410S), FITC rabbit anti-goat IgG (Sigma Aldrich; F2016), and DAPI (Thermo Scientific; D1306) for 1 h at room temperature. Following final washing step with PBS, cells were mounted on glass slides with DAKO Fluorescence Mounting Medium (Dako, Glostrup Kommune, Denmark; S3023). The samples were inspected by super resolution laser-scanning confocal imaging system Carl Zeiss LSM 980 (Carl Zeiss, Co., Ltd., Seoul, Republic of Korea) and images of labeled cells were captured with corresponding 20× and 63× objectives [65,66].

### 4.5. Active Site Labeling of Cysteine Cathepsins

**Figure 10:**
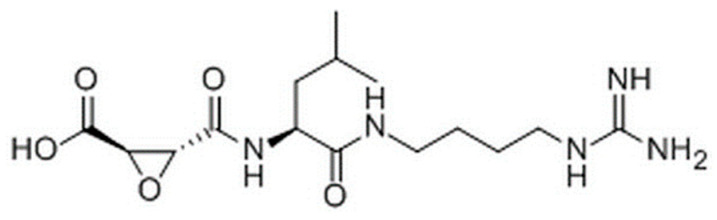
E-64 is an irreversible, potent, and highly selective broad spectrum cysteine proteinase and calpain activation inhibitor. N-[N-(L-3-Trans-carboxirane-2-carbonyl)-L-leucyl]-agmatine. Formula: C_15_H_27_N_5_O_5_.

Cysteine caths in cultured cells were labeled using the activity-based probe DCG04 (Medkoo, Morrisville, NC, USA, CAS, 314263-42-8), which is a biotinylated form of the general cysteine peptidase inhibitor E-64 (sigma Aldrich, 66701-25-5), Figure 10. DCG04 specifically targets and binds to active cysteine peptidases in protein mixtures [67]. The cells were incubated with 10 μM DCG04 at 37 °C for 72 h. Afterward, protein extracts were prepared, and 30 μg of the extract was separated using 12.5% SDS polyacrylamide gels. The proteins were then transferred to a nitrocellulose membrane (Santa Cruz Biotechnology, Dallas, TX, USA, sc-3718). To block the membrane, 3% (on volume basis) bovine serum albumin (BSA) from ThermoFisher Scientific (Waltham, MA, USA) in PBS was used. The membrane was then incubated with streptavidin-horseradish peroxidase in 0.125 μg/mL PBST (BioLegend, San Diego, CA, USA) before being subjected to enhanced chemiluminescence detection.

### 4.6. Pull-Down of DCG04-Labeled Cysteine Cathepsins

To pull down the in vitro-labeled cysteine caths, protein concentrations from cell extracts were measured using the Bradford method (Bradford, 1976) and approximately 400 μg were used [68]. The cellular extracts previously labeled with DCG04 were then diluted with 750 μL of binding buffer (comprising 20 mM sodium acetate at pH 5.5, 150 mM sodium chloride, 0.1% triton X-100, 10 μg/mL E-64, 10 μg/mL leupeptin (Sigma Aldrich, St. Louis, MO, USA, 103476-89-7), and 1 mM PMSF (Abcam, 329-98-6, UK). The resulting mixture was centrifuged at 14,000× *g* for 5 min, and the supernatant was allowed to incubate overnight with 40 μL of settled streptavidin beads at 4 °C. Afterward, the beads were precipitated by centrifugation for 5 min at 3000 rpm; followed by five washes with 20 mM sodium acetate (pH 5.5), 150 mM sodium chloride, and 0.1% triton X-100; and two washes with 10 mM Tris-HCL at pH 6.8 [69]. The settled beads were then mixed with 40 μL of 2× sample buffer and heated for five min at 95 °C [70]. The supernatant obtained was subjected to SDS-PAGE and blotting on a nitrocellulose membrane. The membrane was immunoblotted with antibodies against cath L (dilution 1:2000) from ThermoFisher Scientific, Waltham, MA, USA [51,63].

### 4.7. Cell Viability & Cytotoxicity Test

Cells were seeded in 96 well plates, with 2000 cells per well in 100 μL of medium and left to incubate for 24 h. Following this, the cells were separately exposed to a cath L inhibitor at a concentration of 10 μM and incubated for 24 h before radiation exposure. The viability of the cells after 24 h of treatment was assessed using the 3-(4,5-dimethylthiazol-2-yl)-2 and -5 diphenyltetrazolium bromide (MTT)-based viability assay (EZ4U, Biomedica, Vienna, Austria, Bi-5000). A 20 μL aliquot of EZ4U solution was added to each well, and after 2 h of incubation at 37 °C, the absorbance was measured at 450 nm using a microplate reader (Infinite F200, Tecan, Männedorf, Switzerland) with 620 nm as a reference for the unspecific background values. The entire experimental procedure was performed three times, with triplicates each time [71,72,73].

### 4.8. CRISPR Cas9 and Cath L Gene Knockout

Caco-2 cells were seeded for twenty-four h; when cell confluency had reached 70–90%, caco-2 cells underwent transfection. Two distinct groups were created: one with the CRISPR/Cas9 cath L plasmid (Santa Cruz, sc-419880) and the other with the corresponding control plasmid (Santa Cruz, sc-418922). The transfection process was meticulously executed using the X-tremeGENE™ HP DNA transfection reagent from Roche Diagnostics (catalog Nr. 6366244001, Mannheim, Germany), following the manufacturer’s guidelines with precision.

To initiate the transfection, the X-tremeGENE™ HP DNA transfection reagent, plasmid DNA, and diluent were allowed to reach room temperature with a gentle vortex. Subsequently, the diluent and plasmid DNA were combined and gently mixed in a sterile tube, with the X-tremeGENE™ HP DNA transfection reagent added later to the diluted DNA. The final mixture underwent a 15 min incubation at 25 °C and was then added to caco-2 cells in a dropwise manner, ensuring proper distribution through gentle shaking.

The cells were then incubated with this mixture for 72 h before exposure to varying doses of ionizing radiation [74,75]. This meticulous approach to transfection sets the stage for a comprehensive exploration of the effects of cath L targeting through CRISPR/Cas9 in the context of ionizing radiation exposure on caco-2 cells.

### 4.9. Apoptosis Assay

The Caspase-Glo 3/7 assay (Promega, Madison, IL, USA, G8090) is utilized to gauge the activities of caspase-3 and -7. To conduct the assay, 96-well plates were first seeded with 20,000 cells and then incubated for specified durations in a serum-free medium. Subsequently, the conditioned medium was replaced with the Caspase-Glo reagent, following the guidelines provided by the manufacturer. Luminescence readings were taken using the Infinite F200, Tecan (Männedorf, Switzerland), and the results were reported in relative light units (RLU) [76,77].

### 4.10. Statistical Analysis

The statistical analysis employed a non-parametric *t*-test for the comparison of two groups. In cases involving more than two groups, the choice between one-way ANOVA or two-way ANOVA was made based on the number of independent variables. While the ANOVA F-test yields a significant result, indicating overall group differences, it does not pinpoint specific pairs of means that diverge. To identify such differences among three or more group means, post hoc tests were employed. Following ANOVA, Dunnett’s multiple comparison test was applied to highlight pairs with significant differences. This particular approach compares means from multiple experimental groups against a single control group mean to reveal any notable disparities. To counter the risk of false positives, the Bonferroni test, pioneered by Bonferroni, was implemented. This adjustment ensures the reliability of statistical significance assessments. All statistical analyses were carried out using GraphPad Prism version 6.00 for windows, by GraphPad Software2, San Diego, CA, USA, in conjunction with Microsoft Excel 365. Significance was set at a probability level of *p* < 0.05. The presentation of data followed the format of mean ± standard error (SE). For a comprehensive understanding of statistical parameters specific to each experiment, please refer to the relevant sections or figure legends.

## Data Availability

Data is contained within the article.

## Abbreviations

Caths	Cathepsins
RT	Radiation therapy
Caco-2	Colon Carcinoma Cells
CRISPR	Clustered regularly interspaced short palindromic repeats
CRC	Colorectal cancer
SSBs	Single-strand breaks
DSBs	Double-strand breaks
IR	Ionizing radiation
Gy	Gray
SDS-PAGE	Sodium dodecyl-sulfate polyacrylamide gel electrophoresis
SE	Standard error
DAPI	4′,6-diamidino-2-phenylindole
kDa	Kilodalton
WT	Wild type
DMEM	Dulbecco’s Modified Eagle Medium
ROS	Reactive Oxygen Species
DDR	DNA damage response
NHEJ	Non-homologous end-joining
HR	Homologous recombination
